# Competing World Views, Professional Norms, and Conscience

**DOI:** 10.1007/s10730-025-09562-9

**Published:** 2025-10-15

**Authors:** Ana S. Iltis

**Affiliations:** Wake Forest University, 1834 Wake Forest Rd., Winston-Salem, NC 27106 USA

**Keywords:** Clinical ethics consultation, Conscientious objection, Futility, Life-sustaining interventions, Professional values

## Abstract

Sometimes patients or their legally authorized representatives/surrogate decision makers request interventions that clinicians believe ought not to be provided because, for example, they will merely prolong the life of a patient whose quality of life clinicians deem poor. When such disagreements cannot be resolved, clinicians may request a clinical ethics consultation. Some institutions have policies and some jurisdictions have laws addressing such conflicts, many of which involve procedures that ultimately allow for unilaterally withholding life-sustaining interventions. Autumn Fiester has argued that a radically new approach for addressing such conflicts is needed. I argue that Fiester’s analysis relates closely to and has implications for other debates and key topics in the bioethics literature and raises important questions that merit further exploration. These include the concept of professional values or professional ethics and of values imposition, both of which are important in the literature on conscientious objection.

## Introduction

A major reason for requesting clinical ethics consultation is disagreement over treatment decisions, particularly decisions concerning the use of life-sustaining interventions for patients who have no prospect of recovery or “meaningful” improvement (Milliken et al., 2020; Wasson et al., 2016). Such interventions have been described as futile, medically inappropriate, or non-beneficial, and the terminology remains contested (da Silva Vieira et al., 2021). Sometimes patients or their legally authorized representatives/surrogate decision makers request interventions that clinicians believe ought not to be provided because, for example, they will merely prolong the life of a patient whose quality of life clinicians deem poor. In those cases, clinicians hold that the burdens of treatment exceed its potential benefits (Jecker and Schneiderman, 1993). When such disagreements cannot be resolved, clinicians may request a clinical ethics consultation. Although patients and surrogates also may initiate such requests, this appears to be uncommon (Fox et al., 2022). Some institutions have policies and some jurisdictions have laws addressing such conflicts, many of which involve procedures that ultimately allow for the unilateral withholding life-sustaining interventions. I will refer to these collectively as “futility policies” for the sake of simplicity, although the terminology is far from simple or accurate, and the language itself and its implications have been the subject of significant debate (Bosslet et al., 2017; Brody & Halevy, 1995; da Silva Vieira et al., 2021; Koch, 2023; Kon, 2017; Kopelman, 1995; Misak et al., 2015; Moore, 2023; Nair-Collins, 2015; Nolan, 2020; Rosoff, 2019; Veatch, 2006). The term ‘futility’ often is replaced with other concepts in such policies and laws, such as medically inappropriate care/treatment, nonbeneficial care/treatment, or appropriate care/treatment.

There are numerous reasons for which patients or surrogate decision makers might request life-sustaining interventions even when there is no prospect of recovery. One of those reasons according to Autumn Fiester, is that they hold a worldview she labels as the life-continuation values (LCV) framework. She contrasts this with the best interests values (BIV) framework, which she ascribes to many healthcare professionals and which she argues undergirds futility policies. Fiester argues that these two incommensurable worldviews can result in persistent disagreement about appropriate end of life care. Using futility policies to resolve such conflicts in ways that allow for the unilateral withholding of interventions that would contribute to extending life constitutes, Fiester argues, an imposition of the BIV values system on LCV stakeholders. It is a “tyranny by the majority” (2025, p. 454).

Fiester’s important contribution continues previous work in which she has examined ways in which patients’ and families’ values are marginalized (Fiester, 2015) and her work on methods for ethics consultation that focus on mediation (Fiester, 2013). In this new paper, she engages in ‘moral archeology’ (Fiester, 2015) to identify and articulate the core commitments or tenets of the LCV and BIV frameworks. Her careful exposition of these frameworks reveals values and beliefs that often are not articulated, and that perhaps even people holding them might not always recognize them or describe them as she does. Readers might quibble with some of the details of her account of BIV or LCV. For example, one of the six tenets of LCV is the belief that “Each human life should be extended as long as possible, with maximizing the quantity of life being the ultimate goal of medical interventions” (2025, p. 450). Some people might hold that this is one among several goals of medical interventions that sometimes need to be balanced. LCV adherents balance them differently from how many others do because they attribute more significance to extending life. Or, they might not hold human life must be extended as long as possible but rather that it should be extended much longer than some others might judge. One might question whether the claim that the “LCV worldview is frequently at the root of intractable futility conflicts with BIV stakeholders….” (2025, p. 455) is empirically true. Perhaps there are other value systems or interpersonal dynamics that contribute to many of those conflicts. It is plausible that people who hold something less than or different from the full set of beliefs Fiester includes in the LCV might object to withdrawing life-sustaining interventions and that those objections might persist. (See Weijer, 1998 on resuscitation attempts of Orthodox Jewish patients who are in a persistent vegetative state.) None of that matters for the overall import of Fiester’s analysis and argument as I read it, which shows that:

1. There are incommensurable yet not irrational values that are important to healthcare decision making.^1^ These include the BIV and LCV frameworks.
2. Adherents to BIV are driven by “a discernible values hierarchy” and the BIV is “inherently subjective” (2025, p. 443). Nevertheless, adherents claim that it is value-neutral and they fail to recognize “the perspectival nature of their values framework, viewing it instead as simply the right course of action to achieve the best outcome for the patient given the objective facts” (2025, p. 443). Portraying consensus among physicians or describing a judgment as grounded in the ethics of the profession or the core professional value of doing what is best for patients can lend authority to the BIV framework and be used as a justification for institutionalizing those commitments.
3. “Futility policies” facilitate the imposition of BIV on LCV surrogates.
4. This imposition of BIV on LCV surrogates is ineffectual and wrong, and it disproportionately affects people based on religion or other protected categories. There are least two ways in which disparate impact might arise. First, certain groups, such as people who are deeply religious or members of some other minoritized groups, may be more likely to hold the LCV framework and thus be disproportionately affected by futility policies. Second, comparable cases might be treated differently based on patient or surrogate factors such as race, ethnicity, or educational level. Fiester is not alone in her concerns regarding the disparate impact of such policies and practices and other reasons for which they might be unfair (Pope, 2016; Wojtasiewicz, 2006). One recent study regarding an institutional futility policy found, among other problems that, “The use of the CPP [conscientious practice policy] was inconsistent for different patients, based on the degree to which family members and surrogates persisted in their resistance to limiting medically futile interventions, care providers’ comfort with directly making decisions, and bias towards members of certain groups” (Kaps et al., 2021, p. 48). Rosoff has argued that institutional futility policies are inherently unfair because of “three main irremediable defects in the policy approach: there is the potential for arbitrary decision-making about futility in specific cases; there are structural pre-ordained consequences for ethnic minorities who would be disproportionately affected by the use of these procedures; and the use of rationing justifications to support the use of these policies” (Rosoff, 2013 p. 191). We do not know how often comparable cases are treated differently, but disparate treatment is plausible given other documented instance of it (Gander et al., 2018; Obri et al., 2023; Srinivasan et al., 2023).
5. A radical new approach to addressing such conflicts is needed.

Fiester’s arguments relate closely to and have implications for other debates and key topics in the bioethics literature and raise important questions that merit further exploration. These include the concept of professional values or professional ethics and of values imposition, both of which are important in the literature on conscientious objection.

## Professional Versus Personal Values or Ethical Commitments

The BIV framework that underlies futility policies and that guides their implementation is deeply value-laden and one that people come to hold through a variety of personal beliefs and commitments. As Fiester explains:

Undergirding clinical-decision-making is a discernable values hierarchy that takes a definitive stance on meaningful existence, the good death, appropriate healthcare consumption, proper utilization of resources, and the rightful place of spirituality in medical care. Though this worldview is an articulation of HCPs’ [health care professionals’] own normative priorities—and is therefore inherently subjective—it claims value-neutrality because it is cloaked in the language of patients’ own ‘best interests.’ (Fiester, 2025, p. 443)

Those defending and using futility policies may see them as a means to protecting what is objectively good for patients and therefore appropriate medicine. Sometimes they might refer to these policies as protecting professional integrity or conscience (e.g., Davis, 2008; Daar, 1992). Futility policies enable healthcare professionals to impose their particular worldview, their particular values, and their particular understandings of the good, as captured by the BIV framework, on patients and surrogates who hold a different worldview – the LCV framework.

Fiester’s cogent analysis of these two incommensurable worldviews and the ways in which BIV adherents may impose those beliefs on others through futility policies is particularly important given the explicit and implicit appeals to “bioethical consensus” and related concepts in the clinical ethics literature (Brummett & Watson, 2022; Brummett, 2023a, 2023b) as well as appeals to “accepted professional standards,” professional judgments of what treatment is “beneficial,” “indicated,” or “standard” professional values, professional ethical norms, and professional duties (Schuklenk, 2018; Nelson and Asbhy, 2011; Alexander, 2005; Savulescu, 2006). Such appeals may be used as grounds for articulating various accounts of duties and prohibitions or drawing conclusions about what ought and ought not to be done including, as Fiester discusses, when a life is not worth extending.

There are several reasons to be skeptical about such appeals to an account of professional ethics or values, such as the BIV framework, that is purportedly objective, neutral, or inherent in the profession. One reason, which Fiester does not address, is the reality of moral pluralism within medicine. Many scholars have called into question this notion of a single understanding of professional ethical obligations, duties, standards, norms, or values that is objective or value-neutral or that arises from a shared account of medicine and its goals, values, or norms (see, e.g., Engelhardt, 1996, 2011a, 2011b, 2017; Cherry, 2002, 2018, 2023; Turner, 2003, 2004). Pluralism within medicine and other health professions as well as within bioethics is real, although claims that some authors make in the literature, professional societies’ consensus statements, and other ethics documents may obscure difference. For instance, American Medical Association opinion on Physician Assisted Suicide (Opinion 5.7) says: “Physician-assisted suicide is fundamentally incompatible with the physician’s role as healer, would be difficult or impossible to control, and would pose serious society risks. Instead of engaging in assisted suicide, physicians must aggressively respond to the needs of patients at the end of life.” Anyone reading that might reasonably conclude that physicians in the US regardless of AMA membership (and elsewhere, insofar as they are members of the same profession) would recognize physician assisted suicide (PAS) as wrong and something in which they ought never to engage and ought never to support. Yet, physicians in the US are deeply divided on this today, and we have seen changes in physician attitudes in recent decades (Emanuel et al., 2016; Whitney et al., 2001; Curry et al., 2000; Seale, 2009; Curlin et al., 2008; Lipuma, 2013; Barsness et al., 2020; Piili, 2022). Physicians in some states are legally permitted to participate in PAS and do so. And, some physicians are involved in advocacy work to promote the legalization of PAS. If we were to look not only across time in the US but across geographical locations and cultures, no doubt we would find even more variation in judgments about the permissibility of physician assisted suicide or euthanasia (Sprung et al., 2018; Gutierrez-Castillo et al., 2020; Inbadas 2017; Anh & Huong, 2022).

In medicine there is pluralism about the BIV framework, including disagreement about how to interpret and apply it. Consider the stark example of different claims physicians made regarding the requirements for the ethical practice of medicine when it comes to using life-sustaining interventions in patients with no prospect of recovery in the Quinlan (*In re Quinlan* 70 N.J. 10, 355 A.2d 647 (NJ 1976)) and Cruzan (*Cruzan v. Director, Missouri Department of Health, 110 S. Ct. 2841 (*1990)) cases, on the one hand, and the Wanglie (*In re Helga Wanglie*, Minnesota, Fourth Judicial District (Dist. Ct., Probate Ct. Div) PX-91–283 (1991)) case on the other. Trotter describes the contrast this way:

When Karen Quinlan’s family sought to remove her ventilator in the 1970s, it was because they believed it offered no prospects of further benefit for the permanently vegetative Karen. They were opposed in court by a medical establishment that maintained it had a duty to prolong lives—including even the lives of the permanently unconscious. Healthcare providers in Missouri assumed a similar stance against the family of Nancy Cruzan in 1988. Yet, as Cruzan’s doctors testified in Missouri about their sacred pledge to preserve the life of a permanently vegetative patient, doctors further north in Minnesota formulated a radically contrary argument—that medicine’s venerable ethical tradition precluded them from offering life-prolonging interventions to permanent vegetative state (PVS) patients (Helga Wanglie in this case) because mere prolongation of life without the prospect of regaining consciousness is not a valid goal of medicine. Stunningly, two high-profile cases occurring at the close of the 1980s had different groups of

physicians arguing for precisely the opposite conclusion based on their diverging accounts of professional medical ethics. In both cases, physicians denied the influence of their personal ethical beliefs. Both groups claimed simple loyalty to medicine’s objective, timeworn tradition of professional ethics. And both maintained that the matter should be settled by an appeal to professional values, rather than patient or family preferences. (Trotter, 2007, p. 1)

During the same era and in the same country physicians described radically different accounts of professional medical ethics and the goals and values of medicine, calling into question the legitimacy of appeals to a professional ethic that requires the withholding or withdrawing life-sustaining interventions when patients had no (or little) prospect of recovery.

Another reason for skepticism is the argument that Fiester offers. Regardless of or even in the absence of pluralism within medicine (or another profession), Feister shows that the BIV framework is not a value-neutral or objective account. One justification offered for futility policies is that providing life-sustaining interventions in some circumstances violates professional integrity or professional conscience (e.g., Davis, 2008; Daar, 1992). Fiester’s argument implies that what is portrayed as the “professional conscience” or “professional ethic” whose integrity allegedly is at stake really is a personal conscience or personal account of ethics– it is the product of holding particular values and beliefs that involve “a definitive stance on meaningful existence, the good death, appropriate healthcare consumption, proper utilization of resources, and the rightful place of spirituality in medical care” (Fiester, 2025, p. 443). Fiester shows that healthcare professionals who hold the BIV framework in fact hold one among many value-laden personal beliefs. So, what is at stake is not a professional objective account of what is right versus a set of personal beliefs. Instead, two sets of personal beliefs compete.

On this view, then, futility policies institutionalize and privilege one set of normative commitments and values – the BIV framework – over others. The claim that the BIV framework is the standard view among physicians, or any other professional group, does not change this. It merely sets up the possibility for what Fiester calls the “tyranny of the majority.”

### Beyond the BIV Framework

Fiester’s analysis shows that the BIV framework, although presented as an objectively accurate account of medical obligations and duties and of what is good for patients, is grounded in particular normative judgments, beliefs, and values. So, too, is the LCV framework that some patients and surrogates hold. As a result, what is at stake is not professional versus personal commitments but competing personal commitments. This has important connections to and implications for the literature on conscientious objection and conscience-based refusals of treatment.

Before considering some of the possible implications of Fiester’s argument for conscientious objection, it is important to recognize that what Fiester finds regarding the BIV framework could be true of other norms that are held out as inherent to the profession, or as professional standards grounded in some objective account of what is best for patients. Upon inspection, we might find other commitments that are held out as professional standards but that, like the BIV framework, are held out as inherent to the profession, or that are treated as professional standards grounded in some objective account of what is best for patients, but are grounded in specific values and commitments and reflect particular understandings of the good and a discernable values hierarchy.

Insofar as that is the case, anytime conflicts between those positions and other views that some patient or surrogates hold they also must be described as conflicts between personal beliefs, values, and commitments rather than as conflicts between the professional commitments of healthcare professionals and the personal commitments of patients/surrogates.

## Conscientious Objection

Recognizing that the BIV framework is a personal or subjective normative framework rather than as something inherent in a professional commitment to doing what is objective good for patients recasts conflicts between BIV clinicians and LCV surrogates as conflicts between competing subjective values and norms. One possibile implication of this is that rather than institutionally protecting or privileging the BIV through futility policies, clinicians who hold the BIV framework and want to refuse to provide life-sustaining interventions would have to appeal to their personal consciences to refuse to treat. Decisions about whether they may refuse to treat as well as what options they must disclose to surrogates and whether they must help to identify a willing provider would be guided by conscientious objector/conscientious refusal policies (Ben-Moshe, 2021; Downie et al., 2013; Fry-Bowers, 2020; McConnell, 2021; Shaw & Downie, 2014). It may be that, at least for some physicians, objections to providing life-extending interventions in the circumstances Fiester examines do not rise to the level of conscientious objection. But, insofar as they do, this would be the appropriate mechanism. Assessing whether healthcare professionals’ objections to providing interventions that they believe inappropriately merely prolong life rise to the level of conscientious objections requires assessing whether providing those interventions would undermine their ability to “uphold [their] deepest self-identifying moral beliefs” (Sulmasy, 2008, p. 138). Insofar as it would, this leads to the question of whether healthcare professionals may refuse to provide life-sustaining interventions and if so, under what circumstances. My goal in raising this is not to settle the conscientious objection/refusal debate but to re-situate the conflicts Fiester describes in this frame.

This shift leads to questions about a central theme in the conscientious objection literature, namely the imposition of values. Would physicians who conscientiously object to providing life-sustaining interventions and are permitted to do so be imposing their values on patients or surrogates? Fiester argues that futility policies allow the BIV clinicians to impose their values on LCV surrogates. In the conscientious objection literature, scholars who argue against allowing conscientious refusals, i.e., against allowing healthcare to refuse to provide legal services that fall within the scope of practice), often invoke the language of the imposition of values (e.g., Brummett, 2023a, 2023b; Schuklenk, 2018). Although Fiester and much of the conscientious objection literature invoke the concept of values imposition, little attention is given to articulating the necessary and sufficient conditions for determining that values imposition has occurred. This is an area where more careful analysis is needed.

Concerns over the imposition of values in the conscientious objection literature and in Fiester’s essay focus on professionals imposing their values on patients, but the possibility of values imposition in the other direction is worth considering. In cases in which a refusal is not permitted and clinicians are required to provide the interventions in question, this appears to constitute an imposition of values by patients or surrogates on clinicians. Furthermore, insofar as other people are required to pay for those interventions, we might also ask whether patients or surrogates are imposing their values on them. This question would arise in both private insurance and tax-payer-funded contexts.

Treating clinicians’ objections to providing life-sustaining interventions as matters of conscientious objection will not necessarily make conflicts easier to resolve. There is significant disagreement over whether, when, and how objections to providing particular interventions based on conscience ought to be respected and what such respect would entail (see, e.g., McConnell, 2021; Hershenov, 2021; Brummett, 2023a, 2023b; Symons, 2022; Symons et al., 2024; Sulmasy, 2008; Schuklenk, 2018). However, framing these conflicts in terms of personal commitments might be a more honest approach than one that allows clinicians to hide behind futility policies that privilege their commitments.

Fiester’s work connects to the conscientious objection literature in other ways as well. Scholars who argue that physicians and other healthcare professionals should not be permitted to refrain from providing legal interventions that are within the scope of medicine because they violate their personal beliefs or conscience often appeal to the notion of a professional ethic, norms, values, or ethical standards. These are then contrasted with the personal beliefs or normative commitments of conscientious objectors. For instance, Udo Schuklenk characterizes conscientious objectors this way:

A hallmark of professional judgment is that it is informed exclusively by specialist technical competences and professional values. A conscientious objector insists on overriding what they know a professional judgment would demand of them. They place their personal convictions above their professional obligations. (Schuklenk, 2018, p. 51)

He argues against permitting conscientious refusals this way:

…becoming a doctor is a moral commitment to give priority to ‘the ethical standard of care’ over personal values. Becoming a doctor is, therefore, also ceding authority to professional judgement over personal preference.’ (Rhodes, 2006) ….professionalism and conscientious objection are incompatible. (Schuklenk, 2018, p. 51)

In its Committee on Ethics opinion on conscientious objection and conscience-based refusals to treat, the American College of Obstetrics and Gynecology says:

Professional ethics requires that health be delivered in a way that is respectful of patient autonomy, timely and effectively, evidence based, and nondiscriminatory. By virtue of entering the profession of medicine, physicians accept a set of moral values—and duties—that are central to medical practice. Thus, with professional privileges come professional responsibilities to patients, which must precede a provider’s personal interests. When conscientious refusal conflict with moral obligations that are central to the ethical practice of medicine, ethical care requires either that the physician provide care despite reservations or that there be resources in place to allow the patient to gain access to care in the presence of conscientious refusal. (ACOG, 2007 and 2016)

To describe conflicts as being between personal and professional commitments or values is to assume that there is a professional value or ethical norm at stake that is privileged in some way because it arises from an objective understanding of what is good for patients or from another source that gives that value or norm special status, such as arising from the nature of the profession.

Fiester deflates the depiction of one normative framework that often is portrayed as a “professional norm”—the BIV framework –by demonstrating that it arises from particular personal beliefs, commitments, and conception of the good. One implication of her argument is that conflicts between BIV clinicians and LCV surrogates involve conflicts between competing personal or subjective value frameworks, making it no longer appropriate to characterize them as conflicts between professional norms and surrogates’ personal norms. The same may be true for other norms and values that are held out as “professional norms” but which in fact reflect a values hierarchy and particular understanding of the good. Insofar as that is the case, characterizing healthcare professionals “conscientious objections” as arising from a conflict between a professional judgment or professional moral value and the professional’s personal commitments is mistake. As with the BIV versus LCV conflict, a more appropriate description of the conflict is as one between particular values that have been mistakenly described as professional and personal views (see Hershenov, 2021). It is a conflict between one set of subjective values and another. And, that has implications for the institutional privileging of one over the other.

Someone might respond that conscientious objectors fail to meet the “ethical standard of care” (see Schuklenk, 2018) and that is what is problematic. Attempts to recast the professional norms and values that conflict with healthcare professionals’ personal beliefs in terms of the standard of care will not resolve the problem. Standards of care are informed by, among other things, particular conceptions of the good and rankings of values. To establish the “ethical standard of care” one must first assume particular values and conceptions of the good. For instance, a standard of care that involves withdrawing life-sustaining interventions when a patient has no prospect of recovering significant cognitive function reflects a prioritization of cognitive function over physical life, as Fiester demonstrates. It is only after one accepts the value hierarchy in question that one could articulate that standard as the ethical standard of care. Some of the differences among standards of care we observe, such as the contrast among standards in different countries regarding the withdrawal of life-sustaining interventions, reflect this circumstance (Tanaka et al., 2020). The same might be said of the “standard of care” for children with Trisomy 18 which included only palliative care and not life-saving treatment, typically because their lives were deemed not worth saving because of their anticipated short life span or poor quality of life (Bos et al., 1992; Graham, 2016; Rasmussen et al., 2003).^2^

## Conclusion

Fiester’s analysis sets the stage for substantive re-thinking of policies and practices regarding conflict resolution regarding the use of life-sustaining interventions. It also has important connections to and implications for the conscientious objection literature. Her arguments call on us to investigate and possibly re-think the typical characterization of conflicts between competing understandings of the good or rankings of values that typically are framed as conflicts between professional norms and personal beliefs. There is much work to be done to understand the implications of and concepts that are central to the literature, such as the “imposition of values.” Fiester’s argument makes this work important and compelling.

## Footnotes

1 I suspect that some people would disagree with her claim here and insist that the LCV worldview is irrational.

2 This standard appears to be in flux with clinicians now expressing conflicting attitudes toward offering parents surgical and other aggressive treatment options (Carey, 2021), though disagreement among physicians remains with some judging that Trisomy 18 does not warrant aggressive life-saving treatment (Kochan et al., 2021; Silberberg et al., 2020).
